# Integrating maxillary dentition and 3D facial photo using a modified CAD/CAM facebow

**DOI:** 10.1186/s12903-022-02394-w

**Published:** 2022-08-26

**Authors:** Peiqi Wang, Hui Xu, Rui Gu, Liwei Zhu, Ding Bai, Chaoran Xue

**Affiliations:** State Key Laboratory of Oral Diseases & National Clinical Research Center for Oral Diseases & Department of Orthodontics, West China Hospital of Stomatology, Sichuan University, No. 14, 3rd Section of Renmin Nan Road, Chengdu, 610041 China

**Keywords:** CAD/CAM, 3D stereophotogrammetry, Facebow

## Abstract

**Background:**

Accurate integration of the dentitions with the face is essential in dental clinical practice. Here we introduce a noninvasive and efficient protocol to integrate the digitized maxillary dentition with the three-dimensional (3D) facial photo using a prefabricated modified computer-aided design/computer-aided manufacture (CAD/CAM) facebow.

**Methods:**

To integrate the maxillary dentition with the 3D facial photo, the CAD/CAM facebow protocol was applied to 20 patients by taking a series of 3D facial photos in the clinic and integrating them in the laboratory. The integration accuracy of this protocol was compared with that of a valid 3D computed tomography (CT)-aided protocol concerning translational deviations of the landmarks representing maxillary incisors and maxillary first molars as well as the rotational deviation of the maxillary dentition. The intra- and inter-observer reproducibility was assessed, and the time of clinical operation and laboratory integration was recorded.

**Results:**

This facebow-aided protocol generated 3D fused images with colored faces and high-resolution dentitions, and showed high reproducibility. Compared with the well-established CT-aided protocol, the translational deviations ranged from 0 to 1.196 mm, with mean values ranging from 0.134 to 0.444 mm, and a relatively high integration error was found in the vertical dimension (Z) with a mean ± standard deviation (SD) of 0.379 ± 0.282 mm. Meanwhile, the rotational deviations ranged from 0.020 to 0.930°, with mean values less than 1°, and the most evident deviation was seen in pitch rotation with a mean ± SD of 0.445 ± 0.262°. The workflow took 4.34 ± 0.19 min (mins) for clinical operation and 11.23 ± 0.29 min for laboratory integration.

**Conclusion:**

The present radiation-free protocol with the modified CAD/CAM facebow provided accurate and reproducible transfer of the digitized maxillary dentition to the 3D facial photo with high efficiency.

## Background

Accurate measurement of the spatial relationship between the dentitions and the face is essential in clinical practice from both functional and esthetic perspectives [1]. The occlusal plane (OP) and the mandibular condyle need to be accurately recorded for precise occlusion evaluation, which may otherwise lead to a wrong diagnosis and even subsequent temporomandibular disorders [2]. For orthognathic patients, specifically, inaccurate evaluation of the maxillary arch position impairs the operative outcomes [3]. In addition to functional considerations, in the design and perception of facial esthetics, especially in the fields of orthodontics and prosthodontics, maxillary arch position relative to the facial soft tissue in six degrees of freedom, such as maxillary OP inclination, symmetry of the maxillary arch, and the anteroposterior position of maxillary incisors should be considered [4]. Virtual facebow transfer has been proposed to align the digital maxillary cast to the virtual articulator using standardized extraoral photographs [5]. However, traditional two-dimensional (2D) photos rely much on the head posture and photographing angle and only provide limited information. Therefore, three-dimensional (3D) facial photos with accurately integrated maxillary dentition are important in attempts to make accurate diagnoses and appropriate plans for dental treatment.

The reproduction of the static and dynamic jaw-skull relationship has long been aided by using an articulator, whereas it relies much on the operator experience and can be time-consuming [6], with debatable accuracy and reproducibility [7]. With advancements in technology, computed tomography (CT) scan has been proved to be helpful in the integration of 3D dentitions and 3D facial photos, providing high-resolution dental images and true-color skin texture with accurate tissue spatial relationships. With less chair-side time and a simpler procedure, CT scan constitutes an improved alternative to the articulator [8–14]. However, in cases of teenager maxillofacial deformity screening, in which the patients are young and thus prone to radiation injury; as well as fixed prosthesis, temporomandibular joint diseases, and orthodontic treatment, where the spatial relationship needs to be reproduced repeatedly during evaluation/diagnosis, treatment design, status record, and outcome evaluation, repeated dental CT scan is compromised by concerns of radiation exposure.

**Fig. 1:**
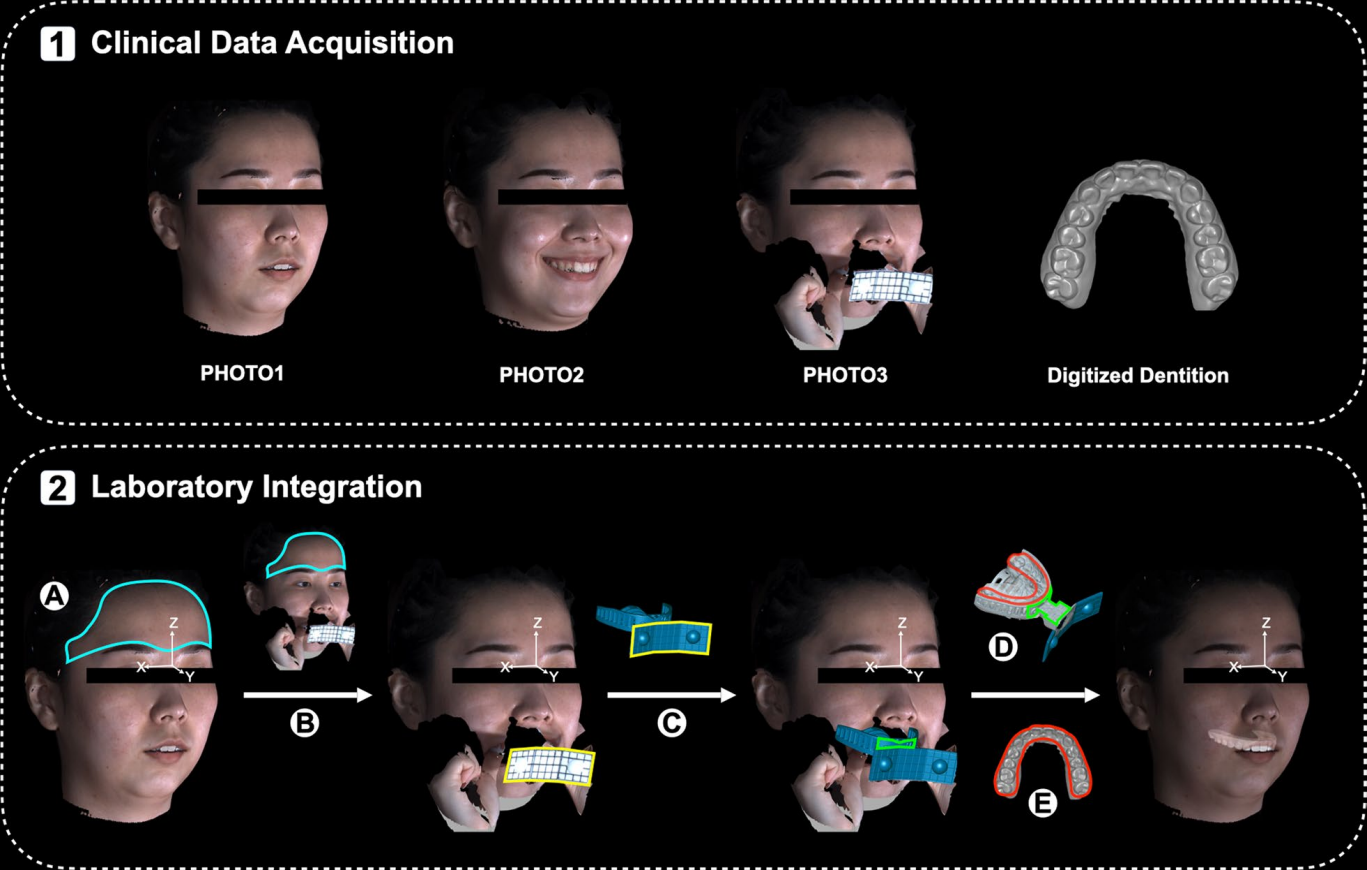
CAD/CAM facebow-guided protocol for integration of the maxillary dentition to the 3D facial photo. Step 1 (upper): clinical data acquisition. Step 2 (lower): laboratory integration. **A** Construction of a facial global reference system on PHOTO1. **B**-**E** Registration of **B** PHOTO3 to PHOTO1 by selecting the facial forehead areas (circled with the blue line), **C** the original facebow geometry to PHOTO3 by selecting the anterior surface of the reflection plate (circled with the yellow line) **D** the scanned facebow with impression to the original facebow geometry by selecting the upper surface of the handle and front end of the tray body (circled with the green line), and **E** the digitized maxillary dentition to the scanned facebow with impression by selecting the maxillary dentition (circled with the red line)

Therefore, non-invasive, easy, and efficient approaches have been developed using fully exposed anterior teeth on the 3D photo for integration [15–17]. To avoid errors and failures resulting from the unclear display of the anterior teeth, special facebow forks have been introduced to locate the maxillary dentition on the 3D face [18–21]. However, the efficiency and effectiveness of device manufacture, information acquisition, and data processing need further verification. This study aimed to establish a noninvasive and time-efficient protocol to transfer the maxillary dentition to the 3D facial photo using a prefabricated modified computer-aided design/computer-aided manufacture (CAD/CAM) facebow (Fig. 1, Step 1 and Step 2).

## Methods

### Subject and criteria

This study was performed from October 2017 to March 2018 at West China Hospital of Stomatology, Sichuan University, Chengdu, China. 20 consecutive surgical-orthodontic patients who required CT for diagnosis were enrolled to prevent excessive use of CT scans. The patients were listed for Le Fort I osteotomy or Le Fort I osteotomy plus bilateral sagittal split ramus osteotomy (BSSRO), of whom 7 were male patients (age range 19–35 years, mean age 26 years) and 13 were female patients (age range 18–34 years, mean age 24 years). Patients with a history of maxillofacial trauma or other congenital anomalies were excluded. Clinical data were collected before orthognathic surgery.

### Design and fabrication of the CAD/CAM facebow

**Fig. 2:**
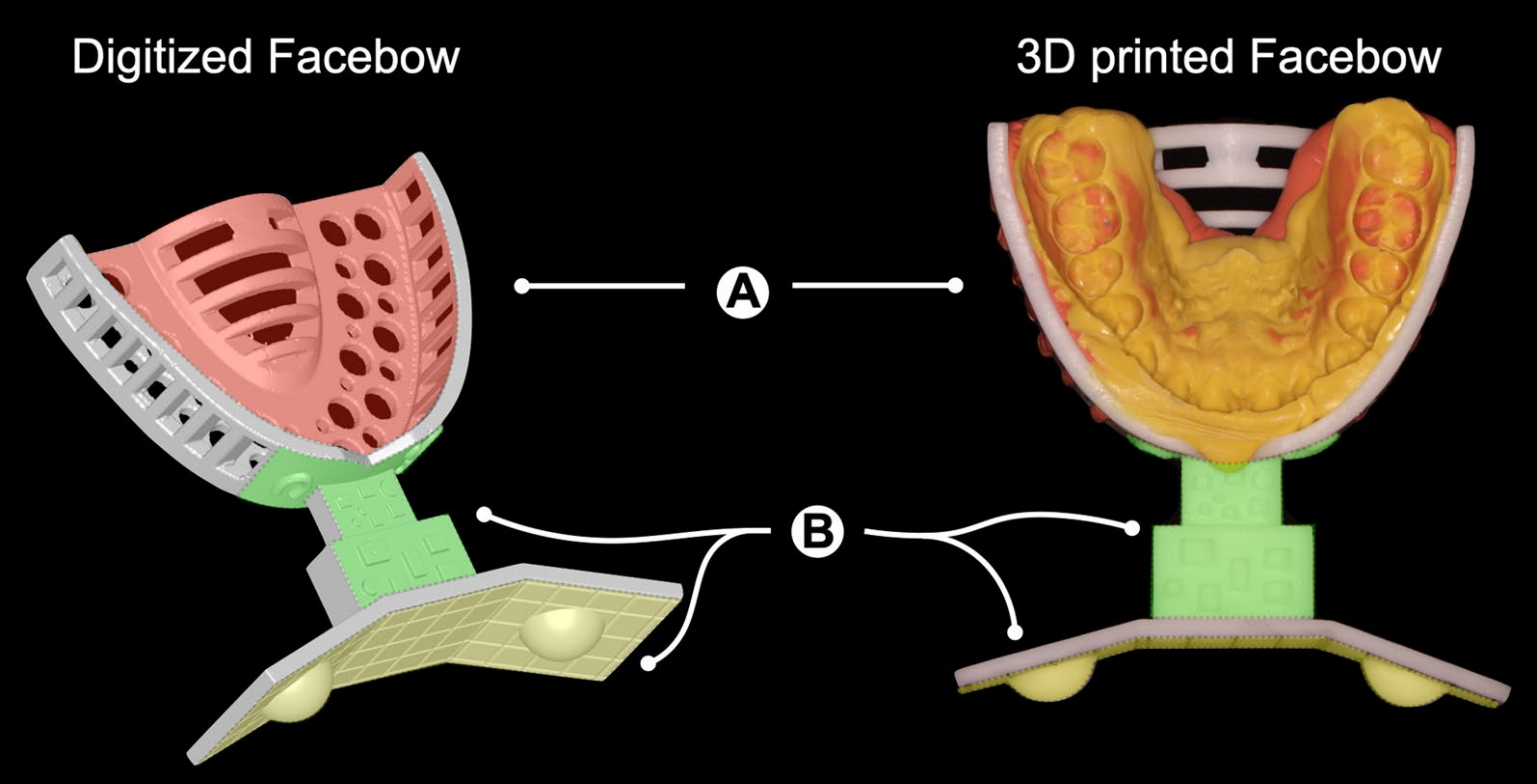
Structure of the CAD/CAM facebow. **A** The intraoral structure: the impression tray for dental impression (marked red). **B** The extraoral structure: the specially designed front end of the tray body and tray handle (marked green) and the reflection plate (marked yellow) for laboratory integration

The CAD/CAM facebow was designed in Geomagic Freeform software (Geomagic, Morrisville, NC, USA). The facebow consisted of two parts: an intraoral part with an impression tray to acquire the dental impression and an extraoral part consisting of the tray handle and a reflection plate (Fig. 2). The extraoral part was specially designed for registration during the laboratory integration (Fig. 2B). To be specific, the impression tray was embossed with protrusions of different shapes on the upper surface of the handle and front end of the tray body for accurate and efficient surface registration (Fig. 2B, green). The reflection plate was composed of a central plane and a lateral plane with a 15° oblique angle in between on each side. Each of the lateral planes was engraved with a hemispherical protrusion (Fig. 2B, yellow). 3D geometries of the facebows in different sizes were exported in .obj files, and the facebows were 3D-printed (Lite600, Uniontech, Shanghai, China) and sterilized prior to use.

### Clinical data acquisition (Fig. 1, step 1)

A facebow of a proper size was selected for each patient. The 3dMDface stereophotogrammetry system (3dMD, Atlanta, USA) was utilized to capture three 3D facial photographs, namely PHOTO1 of the patient with teeth in centric occlusion and soft tissue relaxed, PHOTO2 of the patient in a full smile, and PHOTO3 of the patient with the facebow in place and soft tissue relaxed (Fig. 1, Step 1). Before the capture of PHOTO3, the preliminary putty impression of the two-step putty/wash impression technique had been prepared. After being filled with impression material (Express™ VPS Impression Material, 3 M ESPE; St Paul, MN, USA), the final tray was placed into the patient’s mouth, and PHOTO3 was captured. All 3D photos were exported in .obj files. Chair-side time was recorded. Additionally, the digitized dental casts of each patient were obtained by an intraoral dental scanner (3Shape TRIOS; 3Shape, Copenhagen, Denmark), and the landmarks (UI, A6, B6) on each digitized dentition were selected (definitions seen in Table 1).

**Table 1:** Definitions of the facial global coordinate system, reference landmarks, translational deviations of the landmarks, and rotational deviations of the maxillary dentition

*Facial global coordinate system*	
Origin	The deepest point of the nasal bridge on the facial soft tissue (soft-tissue nasion)
Horizontal plane	Constructed by three landmarks, the most superior point of infraorbital rim on right and left sides and the most superior point of right porion
Mid-sagittal plane	Constructed perpendicular to the horizontal plane, through soft-tissue-nasion and midpoint of both tragus points
Coronal plane	Constructed perpendicular to the horizontal plane and the mid-sagittal plane, passing soft-tissue nasion
Axis	X, transverse axis. Y, sagittal axis. Z, vertical axis
*Landmarks*	
UI	The most mesial point of the tip of the crown of each maxillary central incisor.
A6	The most superior point of buccal groove of the crown of the right first maxillary molar.
B6	The most superior point of buccal groove of the crown of the left first maxillary molar.
*Translational deviations of the landmarks*	
X	Transversal translation of the reference point, “> 0” indicates right movement of the reference point, “< 0” indicates left movement of the reference point.
Y	Sagittal translation of the reference point, “> 0” indicates anterior movement of the reference point, “> 0” indicates posterior movement of the reference point
Z	Vertical translation of the reference point, “> 0” indicates superior movement of the reference point, “> 0” indicates inferior movement of the reference point
*Rotational deviations of maxillary dentition*	
Pitch	Pitch of the maxillary dentition, “> 0” indicates upward rotation of the maxillary dentition in the front around a horizontal axis, “= 0” indicates no rotation of the maxillary dentition on the sagittal plane around a horizontal axis, and “< 0” indicates downward rotation of the maxillary dentition in the front around a horizontal axis, in the global reference system
Roll	Roll of the maxillary dentition, “> 0” indicates rotation of the maxillary dentition around a horizontal axis, up on the left side, “= 0” indicates the maxillary dentition no rotation on the coronal plane around a horizontal axis, and “< 0” indicates rotation of the maxillary dentition around a horizontal axis, down on the left side, in the global reference system
Yaw	Yaw of maxillary dentition, “> 0” indicates left rotation of the maxillary dentition in the front around a vertical axis, “= 0” indicates no rotation of the maxillary dentition on the horizontal plane around a vertical axis, and “< 0” indicates right rotation of the maxillary dentition in the front around a vertical axis, in the global reference system

### **Laboratory process of integration (**Fig. 1, **Step 2)**

#### Establishment of the facial global reference system

With the origin set on the soft tissue nasion point, a facial global reference system was constructed on PHOTO1/PHOTO2 (Fig. 1, Step 2, A, Table 1) using Geomagic Studio 2013 (version 2013; Geomagic, Morrisville, NC, USA).

#### Registration of maxillary dentition to the 3D face

Facebow with the maxillary impression was scanned (3Shape D2000, 3Shape, Copenhagen, Denmark) and exported in .obj file. PHOTO1/PHOTO2 with the facial global reference system was set as a reference, and the Surface Registration approach in Geomagic Studio 2013 was utilized during the whole process of registration. There were four registration steps in total: (1) Registration of the 3D faces (Fig. 1, Step 2, B): PHOTO3 was registered to PHOTO1/PHOTO2 by selecting the forehead area. (2) Registration of the facebow (Fig. 1, Step 2, C): the 3D geometry of the original facebow was registered to PHOTO3 by selecting the anterior surface of the reflection plate. (3) Registration of the impression (Fig. 1, Step 2, D): the scanned facebow with impression was registered to the 3D geometry of the original facebow by selecting the upper surface of the handle and front end of the tray body. (4) Registration of the digitized dentition (Fig. 1, Step 2, E): the digitized maxillary dentition was registered to the scanned facebow with the impression by selecting the maxillary dentition.

In this way, the maxillary dentition was registered to PHOTO1 in the facial global reference system. Laboratory time of the proposed process was recorded.

### **Assessment of reproducibility and accuracy**

#### Assessment of accuracy

The accuracy of the CT-based integration has been well-documented [22], and the CT-aided integration was utilized as the reference standard for accuracy assessment. With data from the spiral CT captured with teeth in centric occlusion and soft tissue relaxed (Philips MX16 EVO CT scanner), 3D reconstruction of the skull with both hard and soft tissues was performed using Mimics (version 10.01; Materialise, Leuven, Belgium).

This reconstructed 3D skull played a similar role to the CAD/CAM facebow in the following procedure. It was registered to PHOTO1/PHOTO2 with the facial reference system using the Surface Registration approach in Geomagic Studio 2013 by selecting the forehead area of the soft tissue, and thus the spatial relationship between soft and hard tissues was accurately recorded. Digitized dental casts with landmarks were then superimposed on the hard tissue of the registered 3D skull. Thus, the maxillary dentition integrated by the CT approach was registered to PHOTO1/PHOTO2 in the facial global reference system.

**Fig. 3:**
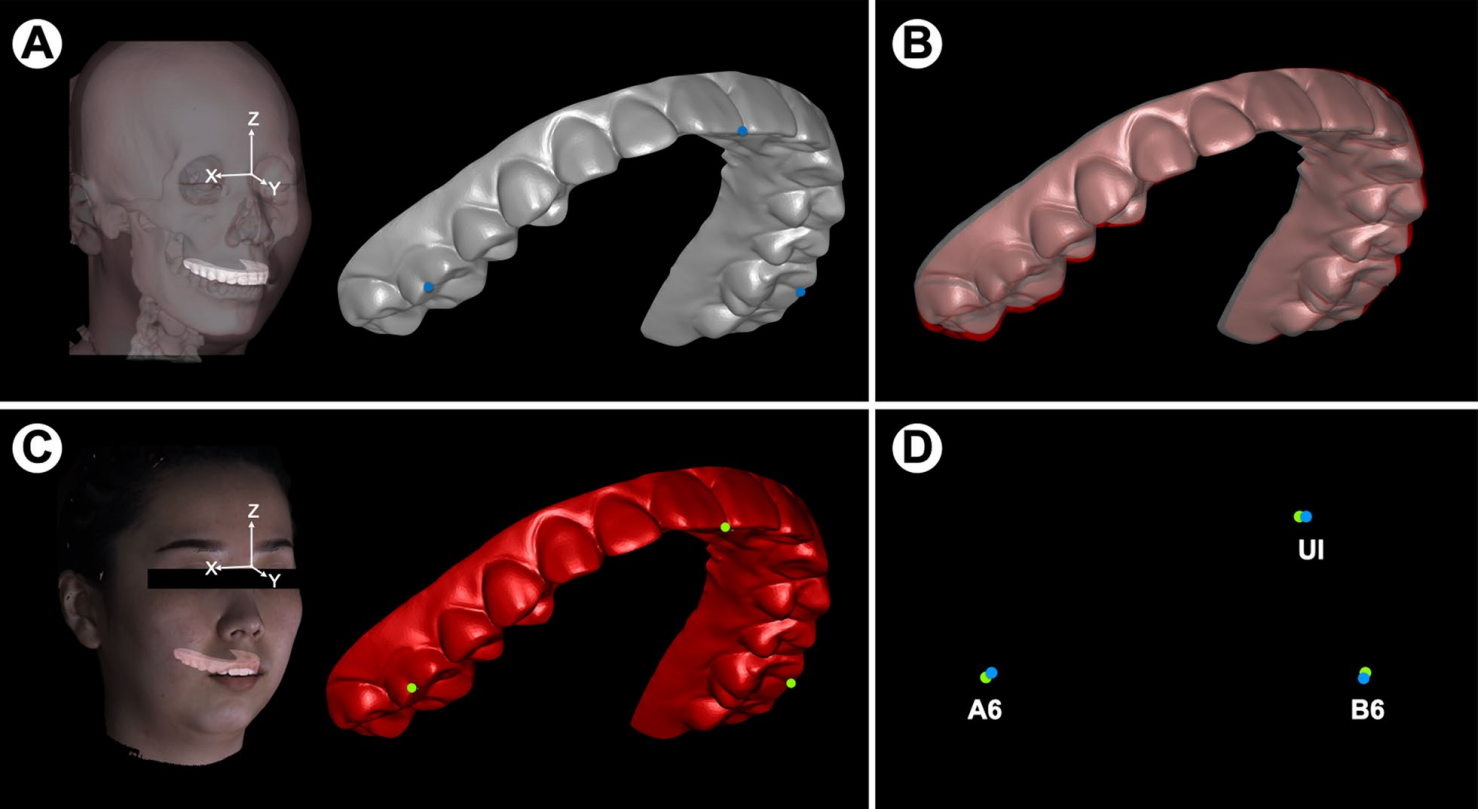
Assessment of accuracy. **A** 3D skull reconstructed from CT data was registered to PHOTO1 with the facial global reference system. The coordinate values of the pre-selected landmarks (blue) on the maxillary dentition were recorded in the CT-integrated position (grey), and the orientations (pitch, roll, yaw) of the maxillary dentition were all set as 0°. **B** By selecting the same region on the dentition, the CT-integrated dentition (grey) with the landmarks “glued” on it was registered to the CAD/CAM facebow-integrated dentition (red). **C** The coordinate values of the landmarks in the position of the CAD/CAM facebow-integrated dentition (green) were recorded. **D** The rotational deviations between the CT-integrated and facebow-integrated dentitions and the translational deviations as represented by differences between the coordinates of the landmarks in the CT-integrated dentition (blue) and the facebow-integrated dentition (green) were automatically recorded

The coordinate values (X, Y, Z) of three landmarks (UI, A6, B6) on the maxillary dentition in the CT-integrated position were recorded, and the orientations (pitch, roll, yaw) of the maxillary dentition were all set as 0° (Fig. 3A). Afterward, the CT-integrated maxillary dentition with the landmarks was registered to the position of the facebow-integrated dentition (Fig. 3B, C). The coordinate values of the landmarks and orientations of the maxillary dentition in the registered position were automatically recorded in Geomagic Studio 2013 (Fig. 3C). In this way, deviations between the facebow protocol and the CT approach could be represented by the positional differences between the CT-integrated dentition and the registered dentition (Fig. 3D). This method eliminated the necessity of repeated positioning of the landmarks and the corresponding errors [23].

#### Assessment of intra- and inter-observer reproducibility

To analyze the intra- and inter-observer reproducibility of the CAD/CAM facebow-guided approach, two independent observers (A and B) performed the integration twice with a 2-week interval between the first and second ones. For these repeated integrations, 12 measurements including the coordinate values (X, Y, Z) of each landmark (UI, A6, B6) and orientations (pitch, roll, yaw) of the maxillary dentition (Table 1) were automatically recorded in the Geomagic Studio as mentioned above.

### Statistical analysis

The observer intra- and inter-observer reproducibility was evaluated using intra-class correlation coefficient (ICC) and Bland-Altman plots. ICC > 0.75 represented excellent agreement beyond chance. Bland-Altman plots were established using the software of Medcalc (version 11.4.2.0; Medcalc Software, Mariakerke, Belgium). Absolute values of all deviations were used for the assessment of accuracy. Mean translational deviations (X, Y, Z) of each landmark (UI, A6, B6) on the maxillary dentitions, and mean rotational deviations (pitch, roll, and yaw) of the maxillary dentitions between the CAD/CAM facebow approach and the valid CT approach were presented. Shapiro-Wilk test showed that some variables were not normally distributed. To compare the deviations above with a clinically acceptable error (1 mm in translation or 1° in orientation), paired *t*-test was applied for normally distributed variables while Wilcoxon matched-pair test was applied for non-normally distributed variables. A level of α = 0.05 was set for significance.

## Results

### Reproducibility of the facebow-aided integration

**Fig. 4:**
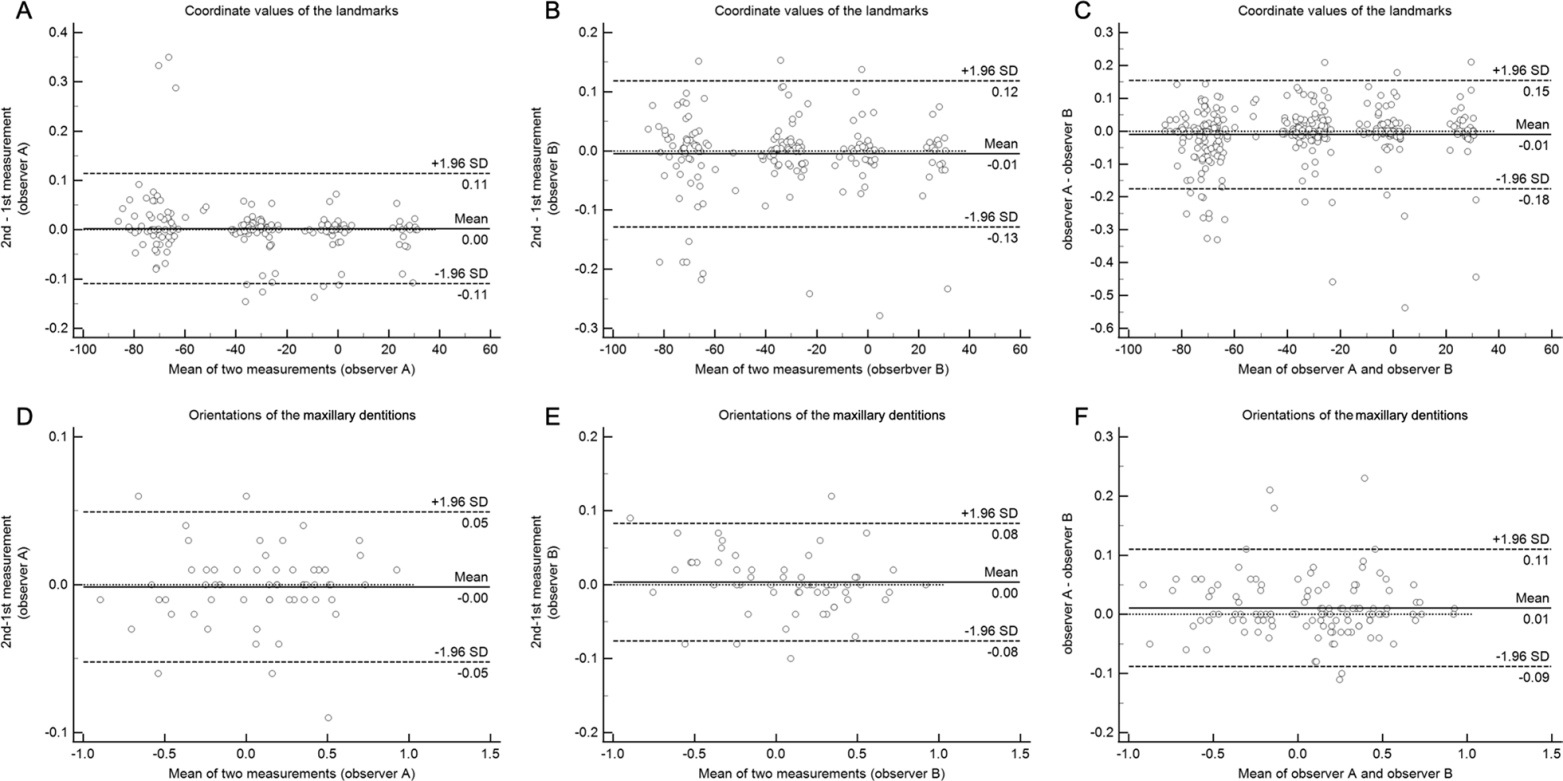
Bland-Altman plots showing the intra- and inter- reproducibility of the CAD/CAM facebow guided protocol. **A–C** Bland-Altman plots of the coordinate values. **D–F** Bland-Altman plots of the orientations

The integration procedure using the CAD/CAM facebow was feasible in all patients and showed high intra- and inter-observer reproducibility as suggested by the ICC values of both planar and rotational descriptors which were much higher than 0.90 (Table 2). Moreover, the Bland-Altman plots (Fig. 4) of the differences between measurements (between operators and time-interval) revealed low mean differences and 95% limits of agreement, indicating high reproducibility.

**Table 2:** Intra- and inter-observer reproducibility of the integration procedure using CAD/CAM facebow-guided protocol^#^

Measurement	Intra-A	Intra-B	Inter-A and B
*Translational deviation*			
X			
UI	0.9998 (0.9994 to 0.9999)	0.9992 (0.9980 to 0.9997)	0.9991 (0.9978 to 0.9997)
A6	0.9999 (0.9998 to 1.0000)	0.9997 (0.9994 to 0.9999)	0.9997 (0.9994 to 0.9999)
B6	0.9999 (0.9997 to 1.0000)	0.9997 (0.9992 to 0.9999)	0.9997 (0.9992 to 0.9999)
Y			
UI	1.0000 (0.9999 to 1.0000)	1.0000 (0.9999 to 1.0000)	0.9999 (0.9998 to 1.0000)
A6	0.9999 (0.9999 to 1.0000)	0.9999 (0.9998 to 1.0000)	0.9999 (0.9998 to 1.0000)
B6	0.9999 (0.9998 to 1.0000)	0.9999 (0.9998 to 1.0000)	0.9999 (0.9997 to 0.9999)
Y			
UI	0.9999 (0.9997 to 1.0000)	0.9999 (0.9998 to 1.0000)	0.9999 (0.9998 to 1.0000)
A6	0.9999 (0.9997 to 1.0000)	0.9999 (0.9998 to 1.0000)	0.9999 (0.9999 to 1.0000)
B6	0.9999 (0.9998 to 1.0000)	0.9999 (0.9997 to 1.0000)	0.9999 (0.9997 to 1.0000)
*Rotational deviation*			
Pitch	0.9976 (0.9940 to 0.9991)	0.995 (0.9873 to 0.9980)	0.9952 (0.9878 to 0.9981)
Roll	0.9977 (0.9942 to 0.9991)	0.9889 (0.9721 to 0.9956)	0.9861 (0.9652 to 0.9945)
Yaw	0.9977 (0.9941 to 0.9991)	0.9967 (0.9917 to 0.9987)	0.9935 (0.9837 to 0.9974)

^#^Results are given as intra-class correlation coefficient (ICC) (95% confidence interval [CI]). Intra-A, intra-observer reproducibility for observer A; Intra-B, intra-observer reproducibility for observer B; Inter-A and B, inter-observer reproducibility between observer A and observer B. CAD/CAM, computer-aided design/computer-aided manufacturing

### Accuracy of the facebow-aided integration

The accuracy of the CAD/CAM facebow-aided protocol was evaluated via absolute translational and rotational deviations from the 3D CT approach (Table 3). As for translational descriptors, the mean deviations of all landmarks were significantly lower than 1 mm (*P* < 0.01). Specifically, deviations in the vertical dimension (Z) were the most evident while those in the transversal direction (X) were the lowest, with means ± SDs of 0.379 ± 0.282 mm and 0.155 ± 0.118 mm, respectively. Among all the landmarks, vertical deviations of A6 and B6 ranged from 0.021 to 1.035 mm and 0.026 to 1.196 mm, respectively, higher than those of UI (ranging from 0.008 to 0.675 mm).

**Table 3:** Translational deviations of the landmarks and rotational deviations of the maxillary dentition between CAD/CAM facebow and CT approaches

Measurement*	Mean	SD	Minimum	Maximum
*Translational deviation* (mm)				
X				
UI	0.192	0.121	0.006	0.430
A6	0.138	0.115	0.000	0.472
B6	0.134	0.110	0.000	0.437
Overall	0.155	0.118	0.000	0.437
Y				
UI	0.248	0.221	0.002	0.697
A6	0.309	0.239	0.009	0.734
B6	0.209	0.170	0.009	0.546
Overall	0.255	0.216	0.002	0.734
Z				
UI	0.353	0.169	0.008	0.675
A6	0.341	0.275	0.021	1.035
B6	0.444	0.358	0.026	1.196
Overall	0.379	0.282	0.008	1.196
*Rotational deviation* (°)				
Pitch	0.445	0.262	0.040	0.930
Roll	0.299	0.199	0.020	0.720
Yaw	0.287	0.150	0.030	0.540

*CAD/CAM* computer-aided design/computer-aided manufacturing; *CT* computed tomography; *SD* standard deviation

*Absolute values were given

In respect of rotational deviations, the mean deviations in pitch, roll, and yaw were significantly lower than 1° (*P* < 0.01), with the means ± SDs of 0.445 ± 0.262°, 0.299 ± 0.199°, 0.287 ± 0.150°, respectively.

### Efficiency of the facebow-aided protocol in clinical operation and laboratory preparation

For clinical data acquisition, the average duration was 4.34 ± 0.19 min (mins). The process of capturing and checking 3D facial photos (including positioning of the final tray to the patient’s mouth) took 1.32 ± 0.15 min, while making the initial tray with the preliminary putty impression and setting the impression material took 3.02 ± 0.15 min, taking up about 69.6% of the clinical time. As for the laboratory integration, the average total time was 11.23 ± 0.29 min. It took 3.00 ± 0.11 min to scan the tray with impression and 8.23 ± 0.30 min to perform the successive registration.

### This protocol provided 3D fused images with colored faces and high-resolution dentitions

**Fig. 5:**
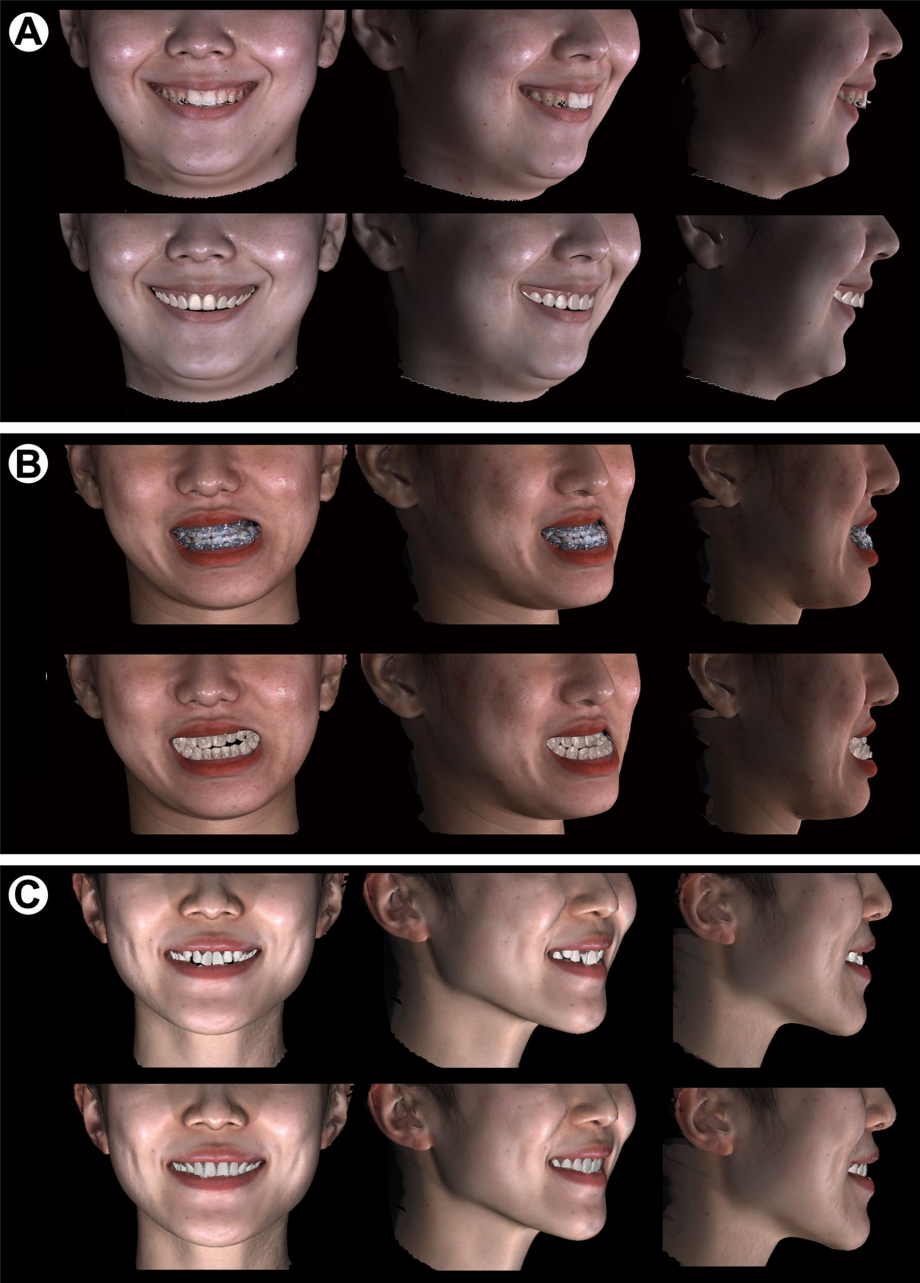
3D images in a full smile. **A, B** 3D facial photograph with original translucent dentition (upper) and 3D fused facial images with clear digitized dentition (lower). **C** 3D fused facial images with clear digitized dentition before treatment (upper) and 3D fused facial images with clear predicted digitized dentition (lower)

With all data registered in the facial global reference system, 3D fused facial photographs with accurately positioned digitized dentitions could be generated, even for teeth with metal brackets (Fig. 5). To re-establish the “red and white esthetics”, teeth and gingiva on the digitized dental casts were colored the same as those on PHOTO2 (Fig. 5). Moreover, this integrating approach, together with techniques such as virtual tooth alignment, virtual prosthodontic planning, and surgery simulation, can provide relatively accurate visual 3D treatment prediction (Fig. 5C).

## Discussion

This study introduced a CAD/CAM facebow to assist the transfer of maxillary dentition to the 3D facial photo with high accuracy and reproducibility in six degrees of freedom. With short-time clinical operation and reduced laboratory preparation, this radiation-free protocol has been proven simple, convenient, and time-efficient for routine clinical use. The approach may be more clinically meaningful since the original digital dentitions can be replaced with the predicted post-treatment digital dentition and soft tissue can be simulated. The estimated results of the treatment can be visually presented to the patients and peers for communication [13, 14].

### Existing protocols to integrate the maxillary dentition and the 3D facial photo

As 3D evaluation provides ample information compared with traditional standardized 2D photos, integration of maxillary dentition and 3D facial photos is important in the diagnosis, treatment, and follow-up evaluation of dental treatment. Integration procedures aided by CT scans have shown satisfactory accuracy [8, 9], but the use is limited due to the radiation exposure, especially in cases requiring repeated integration. In 2008, Rangel et al. proposed to integrate a digital dental cast into a 3D facial picture according to the exposed teeth on the photo [15], and the technique has been utilized and modified since then [16]. Because the anterior teeth were utilized as a reference, any slight deviation on the digital dental images would cause errors in the position of the whole dentition, especially in cases with saliva biofilm or metal brackets where the surfaces of teeth are hard to be recognized [16].

Therefore, integration techniques with transfer units gained extended applications as they dispensed with the matching of the tooth surfaces [18–21]. A transfer unit often comprised an intraoral part to acquire the dental impression and an extraoral structure for registration with the 3D face, and the modifications mainly lay in the design of the extraoral part. For instance, Bechtold et al.[24] introduced a transfer tray containing a facebow fork and a 125-mm-long rod with transmission balls. More recent studies have proposed extraoral components of smaller sizes [18, 20] and the integration has been facilitated with the aid of special targets on the facebow [19]. However, these protocols required full scans of the whole device and thus needed large-volume desktop scanners or long-time scanning with intraoral scanners.

### The special design of the present CAD/CAM facebow

Compared with the CT-aided approach, this proposed procedure poses no radiation and costs less (Table 4), and therefore is more cost-effective and widely applicable. In comparison with the previous integration approaches using transfer units, the present CAD/CAM facebow was embedded with special designs for registration. In addition to the registration targets in the front (reflection plate), there were also patterns for registration on the upper surface of the handle and front end of the tray body (Fig. 2B). In this way, we only needed to scan the upper side of the handle on the scanned facebow to register it to the original facebow whose 3D geometry had been saved in advance (Fig. 1, Step 2, D, Fig. 2 A), thus simplifying and expediting the laboratory integration. Moreover, horizontal and vertical lines as well as the hemispheric protrusions were specially designed on the reflection plate to limit deviations in vertical dimension or roll orientation of the facebow during surface-based registration (Fig. 1, Step 2, C, Fig. 2B).

**Table 4:** Comparison of CT approach, CAD/CAM facebow approach, and traditional facebow to transfer the spatial relationship between the maxillary dentition and the face

	CT approach		CAD/CAM facebow approach		Traditional facebow approach	
Radiation (µSv)	30-1073^#^		0		0	
Cost ($)^##^	CT scan	70	Facebow tray (with impression material)	30	Facebow tray	15
	3D image acquisition	5	3D image acquisition	5	Articulator mounting	85
	Total	75	Total	35	Total	100
Effectiveness	Soft tissue	Yes	Soft tissue	Yes	Soft tissue	No
	Bone	Yes	Bone	No	Bone	No
	Dentition	Yes	Dentition	Yes	Dentition	Yes
	Spatial relationship	Yes	Spatial relationship	Yes	Spatial relationship	Yes
Efficiency (min) ^###^						
Clinical operation	CT scan	1.5–2.5	3D image acquisition	4–4.50	Facebow application	10–15
Laboratory processing	Laboratory reconstruction	2–3	Laboratory integration	11–11.5	Laboratory mounting	40–60
Total		3.5–5.5		15–16		50–75

*CT* computed tomography; *CAD/CAM* computer-aided design/computer-aided manufacturing

^#^Radiation of CT approach is given as effective dose exposure by American Dental Association

^##^Cost of each approach represents the prices charged for each patient in West China Hospital of Stomatology, Sichuan University, Chengdu, China

^###^Efficiency of each approach is given as approximate clinical and laboratory time spent by the same group of experienced operators. The duration of laboratory mounting included time for the plaster to cure. The CBCT scans were taken by 3D Accu-I-tomo (Morita, Japan), the spiral CT scans were taken by MX16 EVO (Philips, Holland), and the time spent on CT scans includes the procedure of patient and machine preparation

The protocol included clinical data acquisition and laboratory integration, average durations of which were 4.34 and 11.23 min, respectively. Specifically, the setting of the impression material took up about 69.6% of the clinical time. Therefore, the one-step impression can be adopted in place of the two-step impression in future practice to accelerate the procedure.

### Accuracy of the CAD/CAM facebow protocol as measured in six degrees of freedom

Although some integration protocols have been suggested, only limited information has been given on their accuracy. Generally, similar to the present study, the accuracy has been represented by measuring the deviations between the virtual facebow-aided integration and the CT record. Lam et al. [18] presented the deviations measured in Euclidean distances: 0.66 mm, 0.58 mm, and 0.26 mm for the maxillary incisors, canines, and first molars, respectively. Further, Li et al. [20] reported the 3D deviations including Euclidean deviations of the landmarks and the rotational deviations of the occlusal plane. To be specific, the deviations of the maxillary incisor, the left first molar, and the right first molar were 1.14 mm, 1.20 mm, and 1.12 mm, respectively, and the mean rotation of the occlusal plane was 1.48°. However, since the dental assessment comprises positional measurements in six degrees of freedom [25], it would be more clinically reliable to specify the accuracy evaluation into measurements in the translational dimensions (transversal, sagittal, vertical translations) and rotational dimensions (pitch, roll, and yaw).

In the present study, the integration accuracy of the CAD/CAM facebow approach was determined using a semiautomatic 3D measuring procedure, incorporating measurements of the translational deviations of the landmarks and the rotational deviations of the maxillary dentition. Overall, the protocol showed satisfactory accuracy, with mean translational deviations ranging from 0.134 to 0.444 mm for all landmarks, and mean rotational deviations ranging from 0.299° to 0.445° for the maxillary dentitions. Interestingly, the vertical deviations were the most evident translational deviations for all landmarks and tended to be higher in the molar region (ranging from 0.021 to 1.196 mm) than in the incisors (ranging from 0.008 to 0.675 mm) (Table 3). Moreover, the pitch of the maxillary dentitions was relatively high compared with roll and yaw (Table 3). Presumably, the subtle errors during superimposition on the reflection plate in the vertical dimension may be magnified along the dentition since the surface registration was performed in the anterior region. However, the exact reason still needs to be explored to further strengthen the integration protocol.

#### Sample selection of the present study

In this study, surgical-orthodontic patients were enrolled for the following reasons: First, these patients required CT for diagnosis, and therefore, excessive CT scans could be avoided. Secondly, aberrances often existed in the position and/or orientation of the maxillary dentition among these patients, providing a better simulation of the clinical application scenarios of the procedure. Moreover, some of the patients wore metal brackets which made the tooth surface hard to recognize, and thus the integration protocol could be stringently tested and trained for higher accuracy.

### Limitations and future expectations of the present protocol

Interestingly, although the reproducibility of the present protocol was high (Table 2; Fig. 4), the translational measurements showed relatively larger intra- and inter-observer biases than the rotational measurements. This could probably be due to the potential errors occurring in the extra step of identifying the landmarks. Therefore, procedures such as automatic landmark determination could be adopted to further strengthen the reproducibility and reliability of the measurement protocol.

It is also worth noting that one of the limitations is that the laboratory integration still took a relatively long time due to the successive registrations. Though clinically acceptable, the duration may be reduced by improving the relevant 3D software and technician/dentist proficiency. Moreover, with the multiple integration steps, the accuracy of the protocol relies much on the quality of 3D images and 3D models. The scanning systems (3Shape TRIOS intraoral scanner and 3dMDface system) utilized in the present study have been terrified to have high accuracy [26–28], but the potential of errors derived from the 3D image/model should be considered when applying protocol with other equipment or software.

Another concern may be focused on the cost of 3D stereophotogrammetry. The 3D faces in the present protocol were acquired with the 3dMD system which was fast and accurate, but bulky and expensive [26].With more low-cost 3D cameras or relative smartphone applications emerging, this protocol would impose fewer economic burdens on hospitals or dental clinics. Moreover, incorporation of the relationship between the two jaws is needed to further strengthen the clinical application of this protocol.

## Conclusion

The modified CAD/CAM facebow-aided protocol provided accurate and reproducible integration of the digitized maxillary dentition and the 3D facial photo with high clinical efficiency.

## Data Availability

All data generated or analyzed during the current study are available from the corresponding author on reasonable request.

## Abbreviations

**OP**: Occlusal plane
**2D**: Two dimension, two dimensional
**3D**: Three dimension, three dimensional
**CT**: Computed tomography
**CAD/CAM**: Computer-aided design/computer-aided manufacture
**SD**: Standard deviation
